# Alzheimer’s disease-like APP processing in wild-type mice identifies synaptic defects as initial steps of disease progression

**DOI:** 10.1186/s13024-016-0070-y

**Published:** 2016-01-12

**Authors:** Mickael Audrain, Romain Fol, Patrick Dutar, Brigitte Potier, Jean-Marie Billard, Julien Flament, Sandro Alves, Marie-Anne Burlot, Gaelle Dufayet-Chaffaud, Alexis-Pierre Bemelmans, Julien Valette, Philippe Hantraye, Nicole Déglon, Nathalie Cartier, Jérome Braudeau

**Affiliations:** INSERM UMR1169, Université Paris-Sud, Université Paris-Saclay, Orsay, 94100 France; Université Paris Descartes, Paris, France; CEA, DSV, I2BM, MIRCen, Fontenay-aux-Roses, 92265 France; INSERM UMR894, Centre de Psychiatrie et Neurosciences, Université Paris Descartes, Sorbonne Paris Cité, Paris, France; INSERM UMS27, Fontenay-aux-Roses 92265, Université Paris-Sud, Université Paris-Saclay, Orsay, 94100 France; CNRS UMR9199, Fontenay-aux-Roses 92265, Université Paris-Sud, Université Paris-Saclay, Orsay, 94100 France; Department of Clinical Neurosciences, Laboratory of Cellular and Molecular Neurotherapies, Lausanne University Hospital, Lausanne, Switzerland; Neuroscience Research Center, Laboratory of Cellular and Molecular Neurotherapies, Lausanne University Hospital, Lausanne, Switzerland

**Keywords:** Alzheimer’s disease early stages, Aβ ratio, Human samples, Animal model, Extrasynaptic NMDAR, Synaptic failure

## Abstract

**Background:**

Alzheimer’s disease (AD) is the most frequent form of dementia in the elderly and no effective treatment is currently available. The mechanisms triggering AD onset and progression are still imperfectly dissected. We aimed at deciphering the modifications occurring *in vivo* during the very early stages of AD, before the development of amyloid deposits, neurofibrillary tangles, neuronal death and inflammation. Most current AD models based on Amyloid Precursor Protein (APP) overproduction beginning from *in utero*, to rapidly reproduce the histological and behavioral features of the disease within a few months, are not appropriate to study the early steps of AD development. As a means to mimic *in vivo* amyloid APP processing closer to the human situation in AD, we used an adeno-associated virus (AAV)-based transfer of human mutant APP and Presenilin 1 (PS1) genes to the hippocampi of two-month-old C57Bl/6 J mice to express human APP, without significant overexpression and to specifically induce its amyloid processing.

**Results:**

The human APP, βCTF and Aβ42/40 ratio were similar to those in hippocampal tissues from AD patients. Three months after injection the murine Tau protein was hyperphosphorylated and rapid synaptic failure occurred characterized by decreased levels of both PSD-95 and metabolites related to neuromodulation, on proton magnetic resonance spectroscopy (^1^H-MRS). Astrocytic GLT-1 transporter levels were lower and the tonic glutamatergic current was stronger on electrophysiological recordings of CA1 hippocampal region, revealing the overstimulation of extrasynaptic N-methyl D-aspartate receptor (NMDAR) which precedes the loss of long-term potentiation (LTP). These modifications were associated with early behavioral impairments in the Open-field, Y-maze and Morris Mater Maze tasks.

**Conclusions:**

Altogether, this demonstrates that an AD-like APP processing, yielding to levels of APP, βCTF and Aβ42/Aβ40 ratio similar to those observed in AD patients, are sufficient to rapidly trigger early steps of the amyloidogenic and Tau pathways *in vivo.* With this strategy, we identified a sequence of early events likely to account for disease onset and described a model that may facilitate efforts to decipher the factors triggering AD and to evaluate early neuroprotective strategies.

**Electronic supplementary material:**

The online version of this article (doi:10.1186/s13024-016-0070-y) contains supplementary material, which is available to authorized users.

## Background

Alzheimer’s disease (AD) is characterized by amyloid deposits, intracellular neurofibrillary tangles, neuronal loss and a progressive decline in cognitive function [1, 2]. Much progress has been made towards understanding the physiopathology of the disease, mostly through studies of transgenic mice designed to reproduce, as closely as possible, the histological and behavioral features of AD [3, 4].

AD is multifactorial, but the abnormal processing of Amyloid Precursor Protein (APP) is a key element in its development [5]. The physiological functions of APP are unclear, but it has been shown to play crucial roles in spine density, morphology and plasticity [6]. The injection of anti-APP antibodies into the rat brain is known to induce behavioral impairments [7, 8]. Furthermore, APP knockout mice have very low levels of dendritic complexity [9]. Altogether, this suggests that APP has an important physiological role associated with synaptic plasticity as well as trophic properties. Overexpression of wild-type (WT) APP and various mutant forms has been used as a means to model AD in many transgenic mouse lines [10, 11]. In most of these transgenic strains, the significant increase in APP production beginning *in utero* may trigger consequences that are not likely mimicking the biochemical deficit observed in AD. Interestingly, Saito and coworkers recently described a new APP knock-in model without APP overproduction [12]. This model reproduces the cognitive deficits and amyloid plaques of AD, but unfortunately does not provide information about changes occurring early in the development of the pathology.

If the role of amyloid component is crucial, the role of amyloid plaque deposition in disease development is currently a matter of debate [13]. The presence of plaques is a diagnostic criterion for AD, but several studies have suggested that the accumulation of amyloid deposits may have a protective function [14]. Moreover, an absence of plaque has been reported in patients with familial AD and mutant forms of APP [15], whereas abundant Aβ plaques have been found in brain samples from elderly patients without clinical dementia [16–19]. Plaques appear many years after disease onset and they cannot, therefore, be responsible for the early events in AD development [20]. By contrast, soluble Aβ may play an important role in the synaptic and cognitive impairments observed in the early stages of AD [21]. The use of transgenic models displaying higher levels of APP and cleavage products compared to the human situation and inducing artificial phenotypes in few months is therefore likely to be inappropriate for studies of the initial phases of AD. The levels of Aβ produced in these models are much higher than those observed in patients and may have toxic effects unrelated to the early phases of AD. In addition, the negative outcomes of recent clinical trials have fueled debate about the validity of overexpression models. Indeed, most of the therapeutic strategies previously tried and largely unsuccessful, have been tested in such transgenic models. There is a growing body of evidence suggesting that amyloid plaques and tangles occur late in disease progression. Therefore, the development of pertinent protective or disease-modifying therapeutic strategies based on the decrease of these markers does not seem to fit well [22, 23]. These compelling observations demonstrate the need to develop new alternative models of AD more closely mimicking the human disease and in particular the early events in its development.

The present study is an attempt at developing such an alternative model involving the production, *in vivo,* in the mouse hippocampus, of moderate levels of amyloid derivatives, resembling as closely as possible the pattern of expression observed in the hippocampus of human AD patients to study the consequences of initial amyloid pathway engagement. We used this modelling strategy to analyze the events potentially contributing to AD development before the appearance of late hallmarks of the disease, such as amyloid deposits, neurofibrillary tangles and neuronal death. The injection of AAV vectors carrying mutated forms of human APP and PS1 into the mouse hippocampus led to the stable production [24, 25] of APP, βCTF and Aβ peptides, at levels similar to those observed in the hippocampus from AD patients and significantly lower than those present in the hippocampus of APP/PS1ΔE9 transgenic mice. The data generated demonstrate the importance of the Aβ42/Aβ40 ratio, which has already been identified as a relevant biomarker in AD patients [26], together with early changes in synaptic functions, Tau phosphorylation and cognitive deficits. These modifications were observed in the absence of plaque formation, or any sign of inflammation, atrophy and/or neuronal death. They were nevertheless capable of inducing cellular changes, such as the abnormal activation of extrasynaptic NMDARs and a decrease in the levels of neuromodulation-associated metabolites, causing memory impairment. These results suggest that APP processing in a limited number of neurons, as recently observed in sporadic forms of AD [27], may be sufficient to trigger an impairment of hippocampal-dependent behavior.

## Results

### Injections of the AAV-APP and AAV-PS1 vectors lead to APP and PS1 transgenes expression in the hippocampus of wild-type mice, from one month after injection

**Fig. 1:**
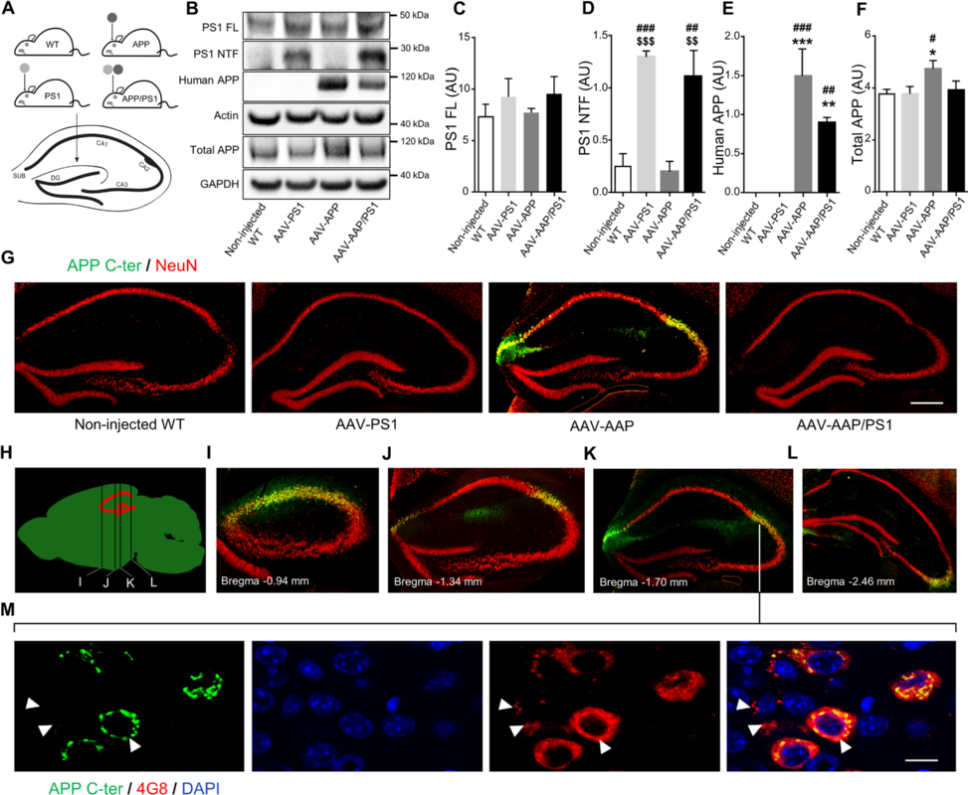
Stereotactic injection of AAV vectors induces the neuronal expression of human APP and PS1 in the hippocampus of C57BL/6 J mice, 1 month after injection. C57Bl/6 J mice (all males) were injected at 8 weeks of age either with AAV-CAG-PS1M146L (AAV-PS1 mice, *n* = 4), AAV-CAG-APPSL (AAV-APP mice, *n* = 4) or both vectors at the same doses as for the other two groups (AAV-APP/PS1 mice, *n* = 4). Non-injected WT mice (*n* = 4) were also analyzed. Mice were killed one month later for analyses. **a** Upper panel: schematic representation of the four groups used. Bottom panel: coronal diagram showing the injection site and the architecture of the mouse hippocampus. **b** Representative western blots showing the expression of PS1 (full length: PS1 FL and N-terminal fragment: PS1 NTF), human APP (6E10 antibody) and total APP (murine + human forms; APP C-ter antibody) confirming transgene expression 1 month after injection. All western blots were performed on the whole hippocampus (TBS-Tx soluble fraction). **c**-**f** Densitometric analyses of western blots showing the expression of PS1 FL (**c**), PS1 NTF (**d**), human APP (**e**) and total APP (**f**) in the four groups hippocampi 1 month after injection. Note that despite the expression of human APP in the AAV-APP/PS1 group, no significant difference in total APP levels was found between non-injected, AAV-PS1 and AAV-APP/PS1 groups. Bars represent means ± SEM and data were normalized with respect to GAPDH. Statistical analysis was performed by one-way ANOVA followed by Tukey’s post-hoc test where ###, *** and $$$ denote *p* < 0.001 versus non-injected WT mice, AAV-PS1 mice and AAV-APP mice respectively. ##, ** and $$ denote *p* < 0.01; # and * denote *p* < 0.05. **g** High magnification of a hippocampal coronal section of each group showing neuronal layers (NeuN antibody, red) and the APP expression (APP C-ter antibody, green). Note that the human APP expression in the AAV-APP/PS1 group (see Fig. 1e) is not sufficient to exceed a threshold detectable by immunohistochemistry. The exogenous APP is essentially detected into two areas: the CA2 and subiculum. Scale bar represents 500 μm. **h** Scheme representing antero-posterior coordinates of coronal sections depicted in (**i**-**l**). **i**-**l** Immunostaining of APP (APP C-ter antibody, green) and NeuN (*red*) at different antero-posterior coordinates in AAV-APP mice coronal sections. **m** Magnified confocal images of an AAV-APP mouse section with double immunofluorescence staining showing the location of APP (APP C-ter antibody, *green*) and the diffusion of Aβ indicated by arrow heads (4G8 antibody, red) in the CA2 layer. *Blue*: DAPI. Scale bar represents 10 μm

We generated AAV vectors encoding human mutant full-length PS1M146L and human mutant APPSL. These vectors were injected bilaterally into the stratum lacunosum of the hippocampus of eight-week-old C57BL/6 J mice. We used the AAVrh.10 capsid, which is known to transduce the central nervous system and the hippocampus efficiently [28]. We studied four groups of animals: non-injected wild-type mice (non-injected WT mice), mice receiving the AAV-CAG-PS1M146L vector (AAV-PS1 mice), mice receiving the AAV-CAG-APPSL vector (AAV-APP mice) and mice receiving a co-injection of both vectors (AAV-APP/PS1 mice, Fig. 1a). To monitor vector-mediated APP and PS1 expression, mice were killed one month after injection. Western blot analysis of whole hippocampus revealed comparable full-length PS1 (PS1 FL) expression in the four groups, despite a trend to increase in AAV-PS1 and AVV-APP/PS1 mice (Fig. 1b and c). Interestingly, 30 kDa PS1 N-terminal fragment (PS1 NTF) was mainly detectable in AAV-PS1 and AAV-APP/PS1 hippocampi (Fig. 1b and d). This PS1 NTF production confirms the endoproteolysis of exogenous PS1 FL which is essential for the formation of the PS1 active site responsible for the formation the Aβ species [29]. AAV-APP and AAV-APP/PS1 mice showed exogenous human APP expression with higher amount in the AAV-APP mice hippocampi (6E10 antibody, Fig. 1b and e). By contrast, the total amount of APP (exogenous human + endogenous murine forms, APP C-ter antibody) did not differ significantly between non-injected WT, AAV-PS1 and AAV-APP/PS1 mice (Fig. 1b and f). Immunohistochemical analyses of hippocampal sections using an APP C-ter antibody revealed a noticeable overexpression of APP only in the AAV-APP mice and observed in the CA2 layer and in the subiculum subfield of the hippocampus (Fig. 1g). Consistent with the result on total APP expression (see Fig. 1f), the human APP expression in the AAV-APP/PS1 group (see Fig. 1e) was not sufficient to be detected by immunohistochemistry. Double-immunostaining with the NeuN antibody confirmed that essentially neurons were transduced by the vectors. No transduction of astrocytes or microglia was observed (data not shown). Analysis of serial anteroposterior coronal sections demonstrated widespread APP C-ter immunoreactivity in the hippocampus from -0.94 mm to -2.46 mm from the Bregma (Fig. 1h-l). Subcellular clustering of human APP was observed in transduced neurons (Fig. 1m). By contrast, staining with the 4G8 antibody, which detects both APP full length and Aβ, yielded a diffuse signal from the intracellular compartment and extracellular medium, suggesting that APP or its cleavage products like Aβ species, may diffuse out of the transduced cells into the adjacent parenchymal space (Fig. 1m).

### Amyloidogenic processing of human APP in AAV-APP/PS1 mice promotes Tau phosphorylation, 3 months after injection

**Fig. 2:**
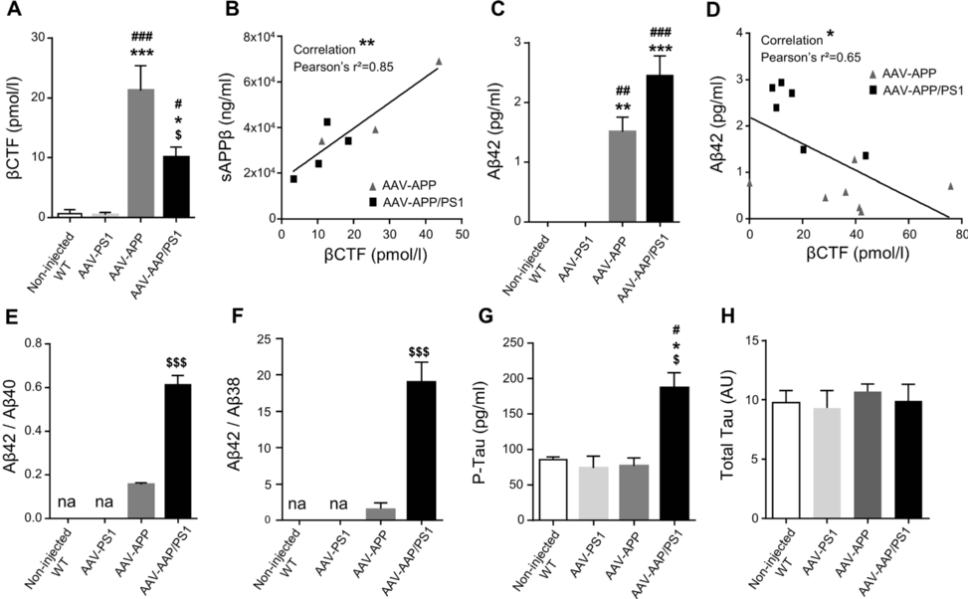
Exogenous human APP is processed following the amyloidogenic pathway, 3 months after injection. C57Bl/6 J mice (all males) were injected at 8 weeks of age either with AAV-CAG-PS1M146L (AAV-PS1 mice), AAV-CAG-APPSL (AAV-APP mice) or both vectors at the same doses as for the other two groups (AAV-APP/PS1 mice, *n* = 7-8 mice per group). Non-injected WT mice (*n* = 4) were also analyzed. Mice were killed three months later for analyses of whole hippocampi. **a** Comparative analysis of TBS-Tx soluble human βCTF levels by ELISA. Note that βCTF levels follow the same pattern of expression than for the human APP in the four different groups (see Fig. 1e). Bars represent means ± SEM. Statistical analysis was performed by one-way ANOVA with Tukey’s post-hoc test where ###, *** denote *p* < 0.001 versus non-injected WT and AAV-PS1 mice. #, * and $ denote *p* < 0.05. **b** Correlation between TBS-Tx soluble human βCTF and sAPPβ levels between AAV-APP and AAV-APP/PS1 mice (*n* = 7). Linear regression analysis confirms the engagement in the amyloidogenic pathway. Correlation analysis was performed with Pearson’s parametric correlation test: **p < 0.01. **c** Comparative analysis of TBS-Tx soluble human Aβ42 levels by MSD immunoassay showing higher levels in AAV-APP/PS1 mice (*n* = 6-8 mice per group). Bars indicate means ± SEM. Statistical analysis was performed by one-way ANOVA followed by Tukey’s post-hoc test where ###, *** denote *p* < 0.001 versus non-injected WT mice and AAV-PS1 mice. ## and ** denote *p* < 0.01. **d** Correlation between TBS-Tx soluble human Aβ42 and βCTF levels between AAV-APP and AAV-APP/PS1 (*n* = 13). Correlation analysis was performed with Pearson’s parametric correlation test: *p < 0.05. **e**-**f** Representation of Aβ42/40 (**e**) and Aβ42/38 (**f**) ratios determined by multiplex MSD immunoassay (*n* = 4 mice per group). Bars indicate means ± SEM. Statistical analysis was performed by one-way ANOVA followed by Tukey’s post-hoc test where $$$ denotes *p* < 0.001 versus AAV-APP mice. na = not applicable. **g** Comparative analysis of TBS-Tx soluble murine phosphorylated Tau protein (AT270, Thr181) by ELISA. Bars indicate means ± SEM. Statistical analysis was performed by one-way ANOVA followed by Tukey’s post-hoc test where #, * and $ denote *p* < 0.05 versus non-injected WT mice, AAV-PS1 mice and AAV-APP mice respectively. **h** Densitometric analyses of western blots showing the expression of the total Tau protein in each group (*n* = 4 mice per group). Bars represent means ± SEM and data were normalized with respect to GAPDH

To confirm the production of human Aβ, we evaluated the processing of human APP in non-injected WT, AAV-PS1, AAV-APP and AAV-APP/PS1 mice three months after vector injection. βCTF, the first cleavage product of the amyloidogenic pathway [30] was quantified by ELISA (Fig. 2a). AAV-APP mice produced larger amounts of human βCTF than non-injected WT, AAV-PS1 and also AAV-APP/PS1 mice (*p* = 0.045). We then quantified the production of the sAPPβ fragment, which is also produced by Bace1 activity, and found a linear relationship between the levels of this fragment and βCTF production (Fig. 2b; Spearman correlation test, r^2^ = 0.85, *p* = 0.0034). Next, we evaluated Bace1 levels and activity. We did not observe any modification between the four groups (data not shown). As observed for the human βCTF, no human Aβ42 was detected in non-injected WT and AAV-PS1 mice. Interestingly, larger amounts of Aβ42 were detected in AAV-APP/PS1 mice than in AAV-APP mice (Fig. 2c), and there was an inverse correlation between βCTF and Aβ42 levels between AAV-APP and AAV-APP/PS1 mice (Fig. 2d; Spearman correlation test, r^2^ = 0.65, *p* = 0.03). These results suggest that, in AAV-APP/PS1 mice, human βCTF is cleaved by the exogenous PS1 to produce larger amounts of Aβ42 peptide. This production/diffusion was restricted to the hippocampus and was not observe in cortical areas (data not shown). We then investigated the ratios of the amounts of the various Aβ species. Aβ42/Aβ40 and Aβ42/Aβ38 ratios were significantly higher in AAV-APP/PS1 compared to AAV-APP mice (Fig. 2e and f; *p* < 0.001). Despite production of both Aβ42 and Aβ40, no amyloid deposit was detected in the cortex and hippocampus of AAV-PS1, AAV-APP or AAV-APP/PS1 mice by staining with either Thioflavin S, Congo red or other amyloid antibodies (data not shown). We also carried out immunostaining for GFAP and Iba1 to analyze the potential astrocytic (GFAP) and microglial (Iba1) responses induced by amyloid APP processing. No recruitment of astrocytes or microglia, or change in cellular morphology suggestive of activation process was observed three months after injection (data not shown). We then investigated the potential impact of the amyloidogenic pathway on phosphorylation of the endogenous Tau protein. ELISA (AT270, Thr181) demonstrated significant increase in Tau phosphorylation in AAV-APP/PS1 mice three months after injection compared to non-injected WT, AAV-PS1 and AAV-APP mice (Fig. 2g; *p* = 0.04). By contrast, no modification of total Tau amount was observed in these four groups suggesting a modification of Tau kinase/phosphatase balance in AAV-APP/PS1 mice (Fig. 2h).

### Similar levels of human APP and amyloid derivatives are observed in the hippocampus of AAV-APP/PS1 mice and AD patients

**Fig. 3:**
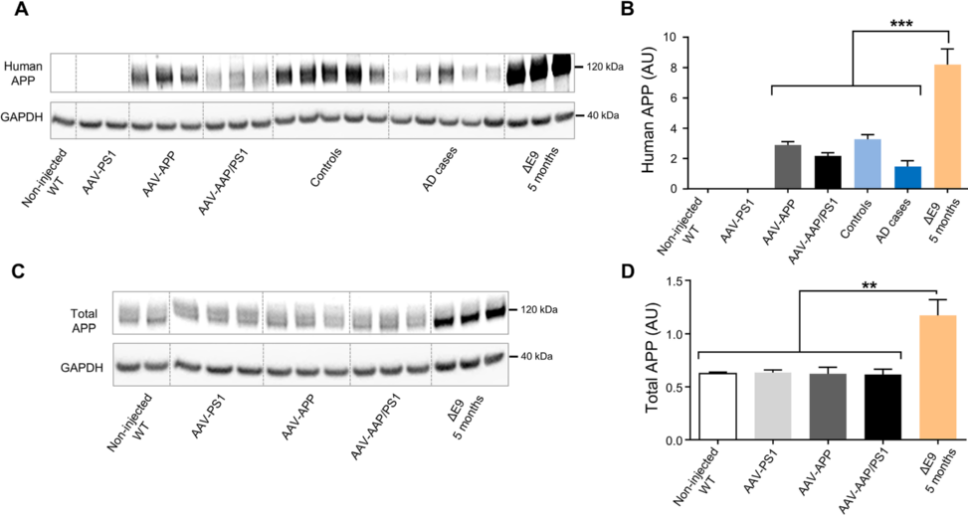
AAV-APP/PS1 mice show a production of human APP similar to AD patients, 3 months after injection. C57Bl/6 J mice (all males) were injected at 8 weeks of age either with AAV-CAG-PS1M146L (AAV-PS1 mice, *n* = 4), AAV-CAG-APPSL (AAV-APP mice, *n* = 4) or both vectors at the same doses as for the other two groups (AAV-APP/PS1 mice, *n* = 4). Non-injected WT mice (*n* = 4) and transgenic APP/PS1ΔE9 mice were also used and all animals were killed at 5 months of age. Human samples were obtained from late-onset AD cases (Braak 6, Thal 5) and age-matched controls. The hippocampus was the structure analyzed for all samples. **a** Representative western blot of human APP (6E10 antibody) between AAV injected mice (*n* = 3 per group), human samples (*n* = 5 per group) and transgenic APP/PS1ΔE9 mice (*n* = 3). **b** Densitometric analyses of the antibody immunoreactivity shown in panel (**a**). Bars represent means ± SEM and data were normalized with respect to GAPDH. Statistical analysis was performed by one-way ANOVA with Tukey’s post-hoc test: ***p < 0.001. Note that AAV-APP/PS1 mice and human AD cases have similar levels. **c** Representative western blot of total APP (APP C-ter antibody) between non-injected WT, AAV injected and APP/PS1ΔE9 mice. **d** Densitometric analyses of the antibody immunoreactivity shown in panel **c**. Bars represent means ± SEM and data were normalized with respect to GAPDH. Statistical analysis was performed by one-way ANOVA with Tukey’s post-hoc test: **p < 0.01

**Fig. 4:**
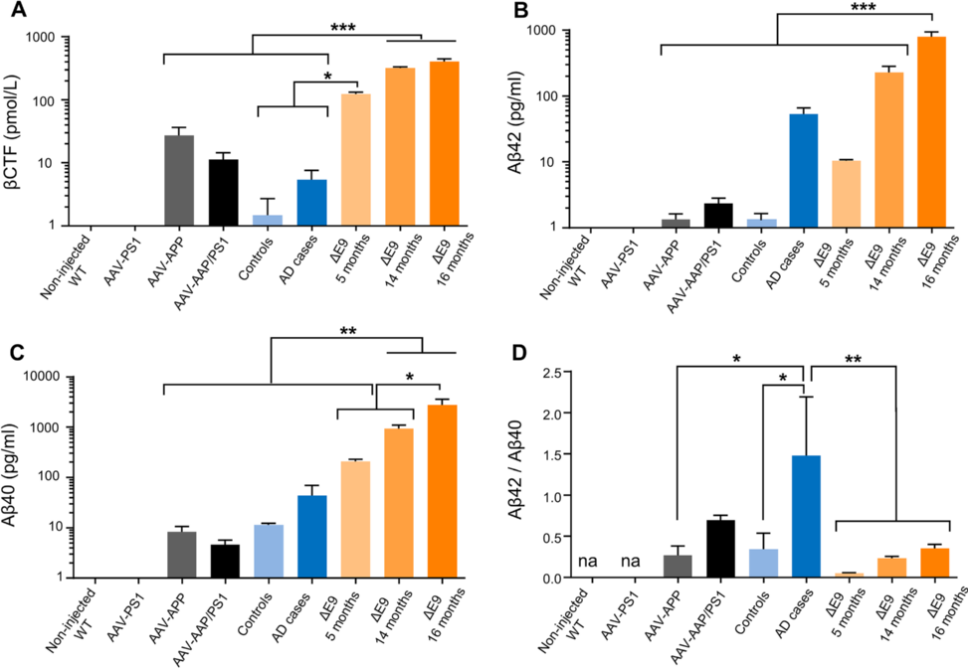
AAV-APP/PS1 mice show a production of amyloid derivatives similar to AD patients, 3 months after injection. C57Bl/6 J mice (all males) were injected at 8 weeks of age either with AAV-CAG-PS1M146L (AAV-PS1 mice, *n* = 4), AAV-CAG-APPSL (AAV-APP mice, *n* = 4) or both vectors at the same doses as for the other two groups (AAV-APP/PS1 mice, *n* = 4). Non-injected WT mice (*n* = 4) and transgenic APP/PS1ΔE9 mice at 5 (*n* = 3), 14 (*n* = 8) and 16 (*n* = 8) months of age were also used. Human samples were obtained from late-onset AD cases (Braak 6, Thal 5) and age-matched controls. The hippocampus was the structure analyzed for all samples. **a** Comparative analysis of TBS-Tx soluble human βCTF levels by ELISA. Statistical analysis was performed by one-way ANOVA with Tukey’s post-hoc test: ***p < 0.001. A logarithmic scale was used. Note that AAV-APP/PS1 mice and human AD cases have similar levels. **b**-**c** Quantification (6E10 based MSD immunoassay detecting human Aβ species) of TBS-Tx soluble human Aβ42 (**b**) and Aβ40 (**c**). Statistical analysis was performed by one-way ANOVA with Tukey’s post-hoc test: ***p < 0.001, **p < 0.01. Note that AAV-APP/PS1 injected mice show higher levels of Aβ42 compared to human controls and reduced levels compared to late stage human cases. **d** Representation of the Aβ42/40 ratio. Note that no significant difference was detectable between AAV-APP/PS1 mice and human AD cases

We compared the levels of APP and amyloid derivatives in non-injected WT, AAV-PS1, AAV-APP and AAV-APP/PS1 mice (3 months after injection i.e. in five-month-old mice) with those present in the hippocampus of human controls and AD patients (five age-matched controls + five AD Braak 6/Thal 5 patients) and in APP/PS1ΔE9 transgenic mice [31]. Human APP content was found to be lower in AD cases than in age-matched controls, even after normalization for neuronal NeuN marker expression (*p* = 0.03), suggesting a higher rate of APP processing in sporadic forms of AD (Fig. 3a and b; Additional file 1: Figure S1). The amount of human APP present in the hippocampus of AAV-APP/PS1 mice was similar to that observed in the hippocampus of AD patients. By contrast, much larger amounts of human APP were found in five-month-old APP/PS1ΔE9 mice (Fig. 3a and b, *p* < 0.0001). Interestingly, total amount of APP was not significantly modified by virus injection compared to non-injected WT mice and in contrast to 5 months old APP/PS1ΔE9 who displayed a two-fold accumulation (*p* = 0.0011, Fig. 3c and d). We then evaluated metabolites released by the amyloidogenic pathway into the hippocampus of mice receiving AAV injections and compared it to those found in APP/PS1ΔE9 transgenic mice at different ages and human AD patients. We used 14 and 16-month-old APP/PS1ΔE9 mice because we detected behavioral deficits in these mice from 14-month-old of age (internal data). βCTF levels which tend to increase in AD patients compared to human controls, were similar in AAV-APP/PS1 mice and in AD patients. Interestingly, these levels were significantly lower compared to both 14 and 16-month-old APP/PS1ΔE9 mice (Fig. 4a; *p* < 0.0001). In AAV-APP/PS1 injected mice, a tendency towards larger amounts of Aβ42 than in human controls was observed, but Aβ42 levels were lower than those in patients with advanced AD and APP/PS1ΔE9 mice at 5 months of age (Fig. 4b). Furthermore, Aβ40 levels were significantly higher in APP/PS1ΔE9 mice compared to other groups and increased with aging in these animals (Fig. 4c). Numerous studies have established the ratio of Aß42 to Aß40 as an important factor in determining the toxicity of Aβ in vivo [32]. We confirmed that Aβ42/Aβ40 ratios were significantly different between human controls and AD patients (Fig. 4d). Interestingly, these ratios were similar between AD patients and AAV-APP/PS1 mice, but significantly different in APP/PS1ΔE9 mice, which had a particularly low Aβ42/Aβ40 ratio. Together, these results indicate that co- injection of the AAV-APP and AAV-PS1 vectors into the hippocampus of normal mice leads to an overall pattern of amyloid processing close to that observed in the hippocampus of AD patients and contrasts to that of APP/PS1ΔE9 mice. Based on results obtained during analysis of APP processing and because AAV-PS1 mice and non-injected WT mice appeared similar in all analyses (see Figs. 1, 2, 3 and 4), we decided to use AAV-PS1 mice as an injected control group for further characterizations.

### NMR spectroscopy shows changes to the neurochemical profile of the hippocampus in AAV-APP/PS1 mice

**Fig. 5:**
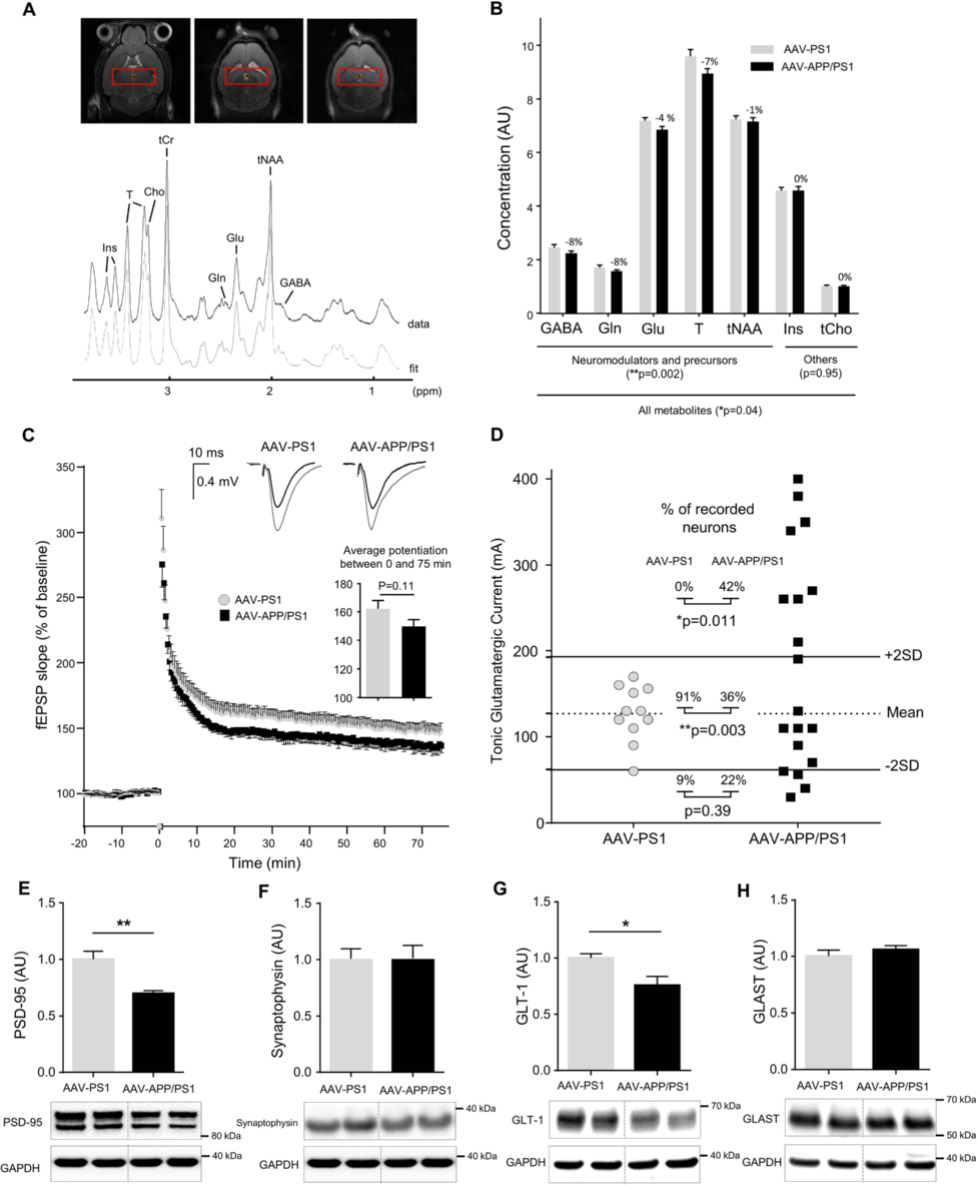
AAV-APP/PS1 mice present synaptic defects, 3 months after injection. C57Bl/6 J mice (all males) were injected at 8 weeks of age either with AAV-CAG-PS1M146L (AAV-PS1 mice used as an injected control group, *n* = 10) or AAV-CAG-APPSL + AAV-CAG-PS1M146L (AAV-APP/PS1 mice, *n* = 10). Mice were used for *in-vivo* (**a**-**b**) and *ex-vivo* (**c**-**d**) recording three months later. **a** *Top* panel: localization of the spectroscopic volume of 7.2 mm x 2 mm x 2.6 mm encompassing both. Hippocampi of each mouse. Bottom panel: representative ^1^H-MR spectrum acquired from the hippocampus of a mouse 3 months after injection. **b** Concentrations of seven metabolites were determined from spectra: GABA, Gln, Glu, T, tNAA, Ins and tCho. Macromolecules and lipids were not included in the study and the values obtained were expressed as ratios relative to tCr (*n* = 10-11 per group). Bars represent means ± SEM. Statistical analysis was performed by two-way ANOVA with experimental group and metabolites as effects. Note that metabolite levels were significantly lower in AAV-APP/PS1 mice (experimental group effect: *p* = 0.04). A selective analysis of metabolites linked to neuromodulation and precursors (GABA, Gln, Glu, T, tNAA) increased the significance of the difference between both groups (experimental group effect: *p* = 0.002). **c** Long-term potentiation (LTP) over 75 min induced by high-frequency stimulation. The inset graphs represent for each group an example of unit field excitatory postsynaptic potentials (fEPSP) before (black line) and after (gray line) LTP induction. The inset histogram shows average potentiation. Bars represent means ± SEM (*n* = 15-16 slices from *n* = 10 mice per group). Statistical analysis was performed with Student’s t-test. **d** Scatter diagram showing the tonic glutamatergic current recorded at a holding potential of +40 mV (whole cell patch-clamp of CA1 pyramidal cells, *n* = 11-19/group from *n* = 10 mice per group). Normal response was characterized in a range comprised between AAV-PS1 mean +/- 2SD. Analysis of AAV-APP/PS1 profile revealed a decrease of number of neurons with normal response (Chi^2^ test: *p* = 0.003) in association with an increase of neurons with an high level of Tonic glutamatergic current (Chi^2^ test: *p* = 0.011) suggesting this current was stronger in the AAV-APP/PS1 group. **e**-**h** Western blot analysis of PSD-95, Synaptophysin, GLT-1 and GLAST. Bars represent means ± SEM and data were normalized with respect to GAPDH. Student’s t-test was used for statistical analysis: *p < 0.05

Proton magnetic resonance spectroscopy (^1^H-MRS) has been shown to detect modifications in AD patients [33]. Using a 11.7 T scanner, we acquired ^1^H-MRS spectra *in vivo* from the right and left hippocampi of AAV-PS1 and AAV-APP/PS1 mice, three months after injection, to evaluate the early consequences of APP processing on hippocampal metabolites. We determined the concentrations of seven metabolites from spectra: GABA, glutamine (Gln), glutamate (Glu), taurine (T), N-acetyl-aspartate + N-acetyl-aspartyl-glutamate (tNAA), myo-inositol (Ins) and glycerophosphocholine + phosphocholine + choline (tCho). Macromolecules and lipids were not included in the study and the values obtained were expressed as ratios relative to creatine + phosphocreatine (tCr) (Fig. 5a). A two-way Anova with repeated measure revealed a group effect (*p* = 0.04). The concentration values obtained were generally lower in AAV-APP/PS1 than in AAV-PS1 mice, in absence of significant individual difference for each metabolite (Group x Metabolite interaction effect (*p* = 0.13)). Among studied metabolites, levels of metabolites related to neuromodulation (GABA, Gln, Glu, T and tNAA) were globally lower in AAV-APP-PS1 mice (Fig. 5b, group effect: *p* = 0.002). By contrast, metabolites not associated with neuromodulation (Ins, tCho) were not affected by co-injection of the AAV-APP and AAV-PS1 vectors. These results suggest that there is a decrease in the global pool of metabolites involved in neurotransmission processes in AAV-APP-PS1 mice. They also rule out the possibility of a single metabolite being entirely responsible for changes, at this stage and in this model. In this way, the absence of increased concentration of glutamate excluded glutamate induced excitotoxicity.

### AAV-APP/PS1 mice present early extrasynaptic deficits

A loss of synaptic contacts in the hippocampus is one of the major early neuropathological findings in AD patients [34]. Moreover, deficiencies in glutamate transport have been shown to be associated with AD [35]. We assessed the functionality of glutamatergic synapses by electrophysiological analyses on hippocampal slices. The long-term potentiation (LTP) of glutamate synaptic transmission was first evaluated in mice three months after injection. A trend towards a decrease in LTP expression following high-frequency stimulation but not theta burst stimulation was found to be linked to the co-expression of APP_SL_ and PS1_M146L_ (Fig. 5c; *p* = 0.11). Beside, we recorded the tonic current generated in CA1 pyramidal cells by the ambient levels of glutamate acting on extrasynaptic NMDA receptors (NMDAR). The amplitude of the tonic current was significantly greater in the AAV-APP/PS1 mice and characterized by a significant dispersion of current amplitude suggesting greater disorganization of the synapse in this population than in the more homogeneous AAV-PS1 or AAV-APP mouse groups (Fig. 5d). Indeed, no difference was observed between the AAV-PS1 and AAV-APP groups (Additional file 2: Figure S2). Synaptic dysfunction occurs early in AD [34] and brains of AD patients have been shown to contain abnormally low levels of synaptic proteins [36]. We compared the levels of the PSD-95 and synaptophysin synaptic markers in the hippocampus of AAV-PS1 and AAV-APP/PS1 mice. PSD-95 levels were significantly lower in AAV-APP/PS1 than AAV-PS1 mice (*p* = 0.007), while synaptophysin was not modified (Fig. 5e and f). Next we investigated the contribution of extrasynaptic glutamate receptor uptake further, by assessing the expression of two astrocytic glutamate transporters, GLT-1 and GLAST. AAV-APP/PS1 mice were found to have significantly lower levels of GLT-1 (*p* = 0.003), with no difference in GLAST levels (Fig. 5g and h).

### AAV-APP/PS1 mice develop early cognitive deficits

**Fig. 6:**
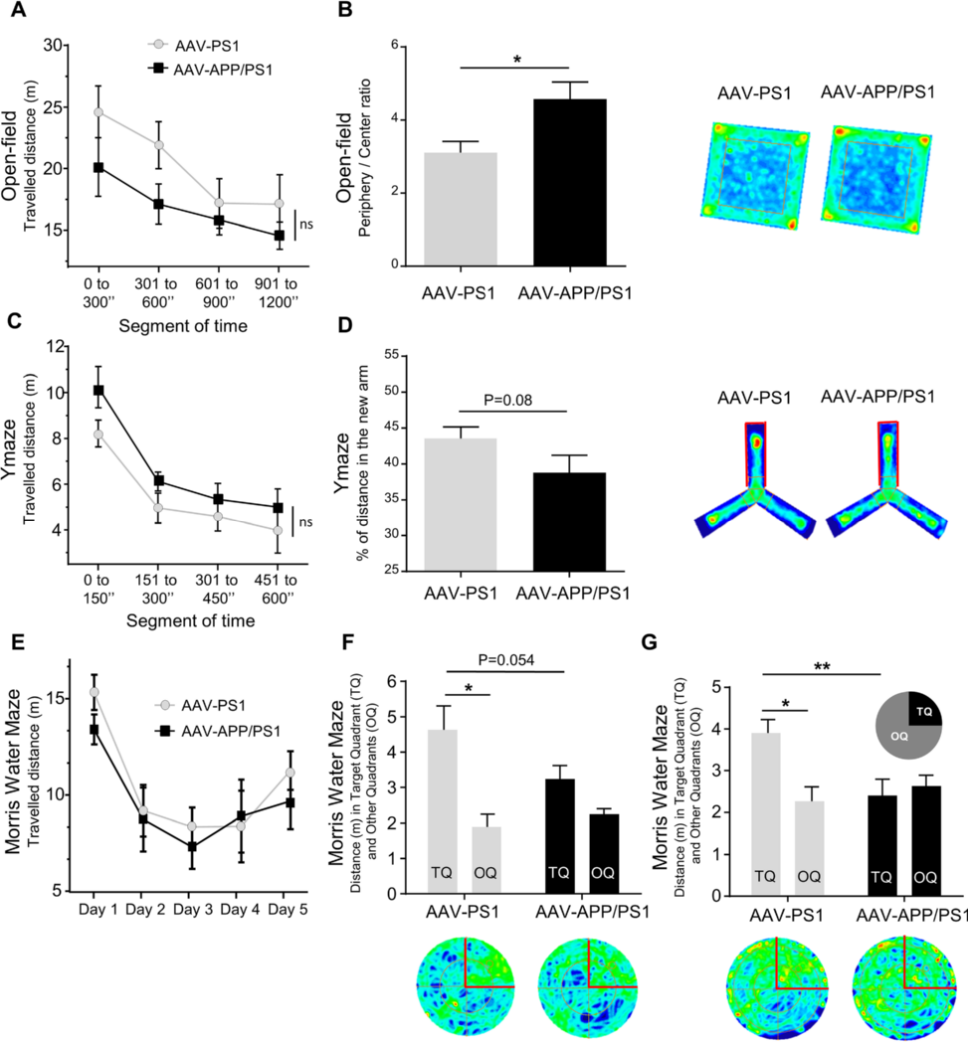
AAV-APP/PS1 mice present memory impairments, 3 months after injection. C57Bl/6 J mice (all males) were injected at 8 weeks of age either with AAV-CAG-PS1M146L (AAV-PS1 mice used as an injected control group, *n* = 8) or AAV-CAG-APPSL + AAV-CAG-PS1M146L (AAV-APP/PS1 mice, *n* = 8) and behavioral analyses were performed three months later. **a** Open-field assay. Travelled distance during the Open-field task showing no significant difference between both groups. **b** *Left* panel: time in periphery/time in center ratio showing a change in emotional behavior when faced to a new environment in the AAV-APP/PS1 group (*n* = 7-8 mice per group). Bars represent means ± SEM. Statistical analysis was performed with Student’s t-test: *p < 0.05. Right panel: group occupancy plots for visualizing the areas in which the animals spent the most time during the test. **c** Y-maze assay. Travelled distance during the Y-Maze task showing no significant difference between both groups during the test session. **d** Left panel: percentage of distance in the new arm showing that AAV-APP/PS1 mice spent less time in the new arm than the other groups (*p* = 0.08; *n* = 7-8 mice per groups). Bars represent means ± SEM. Statistical analysis was performed with Student’s t-test. *Right* panel: group occupancy plots for visualizing the areas in which the animals spent the most time during the test. The arm circled in red is the new arm. **e** Morris Water Maze assay. Travelled distance during the Morris Water Maze task showing no significant difference between both groups during the five training days. **f**-**g** Probe trial performance at 4 h (**f**) and 72 h (**g**) after the last training session. TQ = target quadrant that housed the platform during the training sessions. OQ = mean of distance covered in the other three quadrants. Note that AAV-APP/PS1 mice were impaired in comparison to AAV-PS1 mice confirmed by no preference for the trained target quadrant. Bars represent means ± SEM. A two way ANOVA was used with experimental group and quadrant as main effects: *p < 0.05, **p < 0.01

Most transgenic mouse models of AD reproduce cognitive impairments relevant to the human disease. In most of these models, progressive deficits are associated with excessive Aβ production at an advanced age [3]. We evaluated weather moderate amyloid production in AAV-APP-PS1 mice could be associated with early cognitive dysfunctions. We used AAV-PS1 as control group since previous studies demonstrated that mutant PS1 overexpression in mouse hippocampus did not induce cognitive abnormalities in Morris Water Maze [37] nor in Y-Maze tasks [38], and APP processing profile was similar in WT animals and AAV-PS1 animals (see Figs. 1, 2, 3 and 4). In the Open-field task a decreased distance travelled across time suggests habituation to the novel environment as classically described [39]. Distance travelled during task was similar between AAV-PS1 and AAV-APP/PS1 mice confirming no motor abnormality in our experimental AD mouse model (Fig. 6a). Interestingly, AAV-APP/PS1 mice displayed a much higher periphery/center ratio than AAV-PS1 mice. This difference in behavior may either reflect changes in emotional behavior when faced to a new environment or an increase in anxiety (Fig. 6b; *p* = 0.02). Similarly to Open-field, both groups showed habituation during the Ymaze task, confirmed by a decrease of distance travelled across time (Fig. 6c). No motor impairment was observed in AAV-PS1 and AAV-APP/PS1 mice. Furthermore, a trend towards short term memory impairment was observed in AAV-APP/PS1 mice that spent less time than AAV-PS1 mice exploring the new arm of the maze (Fig. 6d; *p* = 0.08). Finally, we assessed spatial learning and memory, using the Morris Water Maze task. The travelled distance to find the hidden platform was not different between both groups over time (Fig. 6e) with no difference during this learning phase (data not shown). Interestingly, AAV-PS1 mice spent longer in the target quadrant than AAV-APP/PS1 mice (Fig. 5f and g) in both the four-hour (*p* = 0.054) and the 72-h (*p* = 0.0085) probe tests indicating an alteration in memory retention in AAV-APP/PS1 mice. Thus, AAV-PS1 mice displayed a spatial bias in the target quadrant during both the 4-h (target quadrant TQ vs. mean of other quadrants OQ; *p* = 0.031) and 72-h (TQ vs. OQ; *p* = 0.023) probe tests, confirming their preserved spatial memory. By contrast, AAV-APP/PS1 mice did not spend more time in the target quadrant in either the four-hour (TQ vs. OQ; *p* = 0.06) or the 72-h (TQ vs. OQ; *p* = 0.69) probe trial, confirming an absence of efficient memorization of platform position. These data suggest that co-injection of both vectors triggers detectable memory impairment as soon as three months after injection.

## Discussion

Alzheimer’s disease (AD) is a complex condition. To improve our understanding of the physiopathology of the disease and design new therapeutic strategies, it is crucial to get access to the initial phases of its development. We aimed at recapitulating *in vivo* key features that are suspected to account for triggering AD, more specifically an increased Aβ42/Aβ40 ratio, while avoiding major APP overexpression. The importance of the Aβ ratio in the disease onset above their absolute levels has been already proposed [40] and the absence of APP overproduction is increasingly being recognized as an important factor to take into account when modeling AD [12]. Towards this goal, we developed an alternative approach to existing models, involving a single co-administration of two AAV vectors encoding human mutated APP (APP_sl_) and PS1 (PS1_M146L_) in adult wild-type mice hippocampi.

Transgenic mice expressing WT form of the 695-amino acid isoform of APP do not develop AD-like phenotype nor behavioral impairment [41, 42] in contrast to APP751 transgenic mice which have strong learning deficits at 12 months of age [11]. Therefore, the APP751 isoform seems more interesting to trigger an AD-like phenotype. In order to specifically enhance amyloidogenic APP processing, we decided to express the APP751 isoform including the Swedish and London mutations [43]. We simultaneously expressed PS1 with the M146L mutation to increase the specificity of the 42-specific γ-secretase cleavage [44]. Thus, the co-expression of PS1 (PS1M146L) with APP751SL resulted in an increased production of Aβ with an Aβ42/Aβ40 ratio similar to the one observed in AD patients. Interestingly, increase of APP cleavage products was not associated with amyloid deposition, neurofibrillary tangles and neuronal loss.

AAV-APP/PS1 mice display cognitive impairment as early as three months post-injection. These memory deficiencies reflect a global impairment of neuronal activity, as suggested by ^1^H-MRS, showing the levels of metabolites related to neurotransmission expressed in the hippocampus globally reduced. These result highlights that there is an early response of neurons to the APP processing affecting their functionality.

To get further insight in the understanding of this neuronal dysfunction, we used electrophysiological recordings on the CA1 layer to characterize synaptic consequences of moderate soluble Aβ42 production. We observed an enhancement of the tonic glutamatergic current generated in CA1 pyramidal cells of AAV-APP/PS1 mice. Two types of N-methyl D-aspartate receptors (NMDARs) have been described (synaptic and extrasynaptic), the tonic current resulting mostly from the activation of the extrasynaptic subgroup, stimulated by ambient glutamate present outside the synaptic cleft [45, 46]. As we did not observe an increase of glutamate synthesis by ^1^H-MRS, the higher tonic current observed in AAV-APP/PS1 mice may reflect the accumulation of glutamate in the extrasynaptic space. There are two possible explanations for this possible accumulation. First, lower levels of glutamate transporters, such as GLT-1, may result in weaker levels of glutamate uptake by astrocytes [35]. Second, lower levels of scaffolding proteins, such as PSD-95, may lead to the internalization of synaptic NMDARs, resulting in the preferential activation of extrasynaptic ones [47]. AAV-APP/PS1 mice display these features since they express significantly lower levels of GLT-1 and PSD-95. No decrease of GLAST, the second most important glutamate transporter, was observed which might suggest that GLAST expression could be decreased later in the course of the disease [48]. This hypothesis is not surprising, in light of published data about GLT-1 and PSD-95 dysfunctions, which may play an important role in synaptic dysfunction and, thus, in the pathogenesis of AD [49, 50]. To our knowledge, we show for the first time that an impairment of the extrasynaptic compartment could precede sustained alterations of the synaptic compartment associated with LTP deficits, during the early stage of AD progression. We also showed that a subtle loss of neuronal integrity in the hippocampus is sufficient to trigger deleterious effects before actual neuronal death occurs. Despite diffusion properties of Aβ42 [51], no amyloid peptide have been detected outside the hippocampus excluding an Aβ42 direct effect far from its hippocampal production area. By contrast, Aβ altered hippocampus functions could lead to a less efficient communication between hippocampal neurons and connected structures especially cortex and amygdala. This network dysfunction could be responsible of cognitive modifications especially during memory formation [52, 53] or emotional behaviors [54].

In addition to these consequences, AAV-APP/PS1 mice displayed higher levels of Tau phosphorylation (thr181, AT270 antibody) compared to the other groups. It has been reported that soluble Aβ and extrasynaptic NMDAR activation contribute to Tau protein phosphorylation [55, 56]. Our results are consistent with these previous reports. The pattern of Tau phosphorylation was shown to be correlated with the multiple steps of neurofibrillary tangle development [57]. Thr181 immunoreactivity is detected earlier than thr212/Ser214 (AT100 antibody) or ser199/ser202/thr205 (AT8 antibody) immunoreactivity. We could not detect some AT8 or AT100 positive cells (data not shown), suggesting that the modifications observed in AAV-APP/PS1 mice might be compared to early phases of AD in humans. These data suggest that the moderate AD-like APP processing engagement observed in AAV-APP/PS1 mice is sufficient to trigger the Tau pathway engagement.

A particular feature in our experimental strategy is the localized production of human APP and its cleavage products. Contrasting with the ubiquitous overproduction of human APP in mouse transgenic lines, injection of AAV-APP and AAV-PS1 vectors in the stratum lacunosum moleculare region, leads to the transduction of neurons in restricted regions of the hippocampus (CA2 and the subiculum). Interestingly, this pattern of expression may mimic the genomic mosaicism recently described in AD, in which an increase in copy number was observed for the APP gene in a limited number of neurons, in sporadic forms of AD [27]. The presence of rare neurons with APP amplification may be sufficient to trigger a dysregulation of APP processing with aging. Our findings also point to this direction. The second particular feature in our experimental design is the co-injection of the PS1 vector. Indeed, we demonstrated that AAV-APP injection alone was not sufficient to induce hippocampal alteration. Thus, despite the production of βCTF, Aβ40 and Aβ42 in AAV-APP mouse hippocampus, no change in the levels of phosphorylated Tau, PSD-95, GLT-1 or tonic current were observed, compared to the AAV-APP/PS1 group. There are two possible reasons for this difference between the AAV-APP and AAV-APP/PS1 groups. First, APP acts as a trophic factor [58] and an increase in the levels of this molecule may increase neuron viability [59], countering Aβ toxicity. Second, the Aβ42/Aβ40 ratios in the AAV-APP/PS1 group were highly similar to those found in AD patients and different from those of the AAV-APP group or human controls.

Our data raise several questions. This configuration, with no plaque nor tangle and with levels of APP cleavage products close to the human hippocampal condition, is sufficient to induce cognitive impairment three months after injection. It takes about 13 months to obtain an equivalent defect in APP/PS1ΔE9 mice [60]. This delayed cognitive impairment may result from the trophic roles of APP and the low Aβ42/Aβ40 ratio observed in 5- to 16-month-old APP/PS1ΔE9 mice. These findings confirm the greater importance of the Aβ ratio rather than the absolute amounts of Aβ42 and Aβ40 [40] to induce synaptic and extrasynaptic impairments. Finally, neurons may become brittle, leading to cognitive impairments, with defects in emotional behavior and long-term memory.

## Conclusions

This study reports the development of a novel mouse model focusing on the early consequences of the amyloid processing of APP with similarities to human AD cases. Our results support the importance of the Aβ42/Aβ40 ratio to trigger initial neuronal dysfunctions *in vivo*. All mechanisms described here are consistent with memory impairments in the earliest stages of the clinical disease beginning with subtle changes in the efficacy of hippocampal synapses, before amyloid deposition and neuronal loss. It appears that minimal dysregulation of amyloidogenic pathway is sufficient to set up synaptic, and notably extrasynaptic dysfunctions, potential first step towards the full AD phenotype. Using animal models closely mimicking the human disease in terms of APP and APP cleavage products and focusing on early stages of AD development may increase our understanding of disease onset. This model may ultimately make it possible to identify early biomarkers of AD while evaluating relevant neuroprotective therapeutic strategies.

## Methods

### Plasmid design and vector production

We used a double-mutant human APP751 cDNA containing the Swedish and London mutations (codon optimized and containing a Kozak sequence; GeneArt, Life Technologies, Saint Aubin, France), and a human PS1 cDNA containing the M146L mutation (pENTR4-PS1-S182M146L*)*. The APP_SL_ and PS1M146L sequences were cloned in an AAV2 plasmid with CAG promoter to generate the AAV2-CAG-APPSL or -CAG-PS1M146L. AAV vectors were produced as previously described [61], except that the AAV packaging plasmid was replaced with a plasmid construct containing the *rep* gene of AAV2 and the *cap* gene of AAVrh10.

### Human brain samples

Postmortem samples were obtained from brains collected as part of the Brain Donation Program of the GIE-Neuro-CEB Brain Bank. Autopsies were carried out by accredited pathologists, after informed consent had been obtained from the relatives, in accordance with French Bioethics laws. Five hippocampal samples from five patients with sporadic forms of AD (male and female; Braak 6 Thal 5; aged between 69 and 89 years, with a postmortem interval (PMI) of 30 to 59 h) and five hippocampus samples from five age-matched control subjects (male and female, aged between 69 and 92 years, PMI of 6 to 63 h) were used in this study.

### Animals

We used 90 male C57Bl/6 J mice (eight-week-old; SARL JanvierLabs, Le Genest Saint Isle, France) and 19 male APP/PS1ΔE9 mice [31]. All experiments were conducted in accordance with ethical standards and French and European regulations (Directive 2010/63/EU).

### Stereotactic injections of AAVs

Mice were anesthetized by an intraperitoneal injection of ketamine/xylazine (0.1/0.05 g/kg body weight) and placed in a stereotactic frame (Stoelting, Wood Dale, IL, USA). Stereotactic intracerebral injections of AAVs into the hippocampi of both hemispheres were performed, using the following coordinates: antero-posterior: -2 mm, lateral: ± 1 mm, ventral: -2 mm relative to bregma. We injected 2 μl of viral preparation into each site (5 x 10^8^ vg/site and 10^9^ vg/site for AAV-PS1 and AAV-APP vectors, respectively) at a rate of 0.2 μl/min. Four groups were designed, a non-injected wild-type (non-injected WT mice) and three injected groups: AAV-CAG-PS1M146L (AAV-PS1 mice), AAV-CAG-APPSL (AAV-APP mice), AVV-CAG-APPSL + AAV10-CAG-PS1_M146L_ (AAV-APP/PS1 mice).

### Tissue collection and sample preparation

Mice were anesthetized with ketamine/xylazine and transcardially perfused with 20 ml ice-cold phosphate-buffered saline (PBS). One hemisphere was post-fixed by incubation for 48 h in 4 % PFA, cryoprotected in 30 % sucrose in PBS and cut into 40 μm sections with a freezing microtome (Leica) for histological analyses. The contralateral hemisphere was dissected for isolation of the hippocampus and cortex. Samples were homogenized in a lysis buffer (150 mM NaCl and 1 % Triton in Tris-buffered saline, referenced as TBS-Tx) containing phosphatase (Pierce) and protease (Roche) inhibitors and centrifuged for 20 min at 15000 g. The same procedure was applied to human samples (GIE NeuroCEB Brain Bank).

### ELISA

The Aβ extracted was quantified with the MSD Human Aβ42 V-PLEX Kit and the triplex Aβ Peptide Panel 1 (6E10) V-PLEX Kit (Meso Scale Diagnostics, Rockville, USA). βCTF was determined with the IBL Human APP βCTF Assay Kit (IBL International GmbH, Hamburg, Germany). Hyperphosphorylated Tau was determined with the Innogenetics Phospho-Tau 181P kit (Fujirebio Europe, Ghent, Belgium). sAPPβ was determined with the MSD sAPPalpha/sAPPbeta Kit. ELISA was performed according to the kit manufacturer’s instructions in each case.

### Western blotting

Equal amounts of protein (30 μg) were separated by electrophoresis in NuPAGE Bis-Tris Gels (Life Technologies) and transferred to nitrocellulose membranes. The membranes were hybridized with various primary antibodies (APP 6E10, 1/500, Covance; PS1, 1/1000, Millipore; APP C-ter, 1/500, Millipore; Actin, 1/2000, Abcam; GAPDH, 1/1000 Abcam; Total Tau, 1/1000, Santa Cruz; PSD-95, Invitrogen, 1/2000; Synaptophysin, Santa Cruz, 1/200; GAD65, Abcam, 1/2000; GLT-1, 1/1000, Frontier Science; GLAST, 1/1000, Frontier Science; NeuN, Millipore, 1/1000). Various secondary antibodies was also used (ECL Anti-rabbit Horseradish Peroxidase linked, 1/2000, GE Healthcare; ECL Anti-mouse Horseradish Peroxidase linked, 1/2000, GE Healthcare; ECL Anti-rat Horseradish Peroxidase linked, 1/2000, GE Healthcare).

### Immunohistochemistry and image acquisition

Cryosections were washed with 0.25 % Triton in PBS and saturated by incubation (0.25 % Triton in PBS/5 % goat serum). They were then incubated with primary antibodies (APP C-ter, 1/500, Millipore; NeuN-Biotin, 1/1000, Millipore; 4G8-Biotin, 1/1000, Covance). Images were taken with a Nikon Eclipse Ti Microscope or a Leica TCS SP8 confocal microscope and analyzed with ImageJ software (NIH).

### Behavioral assessment

#### Open-field

The apparatus consisted of an open-topped, clear Plexiglas box measuring 50 x 50 x 38 cm placed in a room with controlled dim lighting (25 lux) and constant white noise at 60 dB. The mice were placed in the center of the arena and a video recording was made over a period of five minutes. The behavior of the animals was analyzed from this video. The arena was divided into a central region (20 x 20 cm) and a peripheral region, and the time spent in the center and periphery of the open field was measured. The ratio of time spent in the periphery to that spent in the center was calculated as an index of emotional behavior.

#### Ymaze

The apparatus consisted of three identical arms separated by 120°. Each arm of the Y maze was 37 cm long, and 8 cm wide, with 12.5 cm-high opaque walls. Various extra maze cues were placed on the surrounding walls. One arm of the Y-maze was blocked and the subject was allowed to explore the other two arms for 10 min. The starting position was varied pseudorandomly, between the three arms. The animal was then returned to its home cage. Fifteen minutes later, the mouse was placed in the maze again, this time with all three arms open, and allowed to explore for an additional five minutes. The distance traveled and the number of times the mouse entered each arm were measured both during initial exposure to the maze and during testing.

#### Morris water maze

Experiments were performed in a tank 120 cm in diameter and 50 cm deep, filled with opacified water kept at 21 °C and equipped with a platform 10 cm in diameter, kept submerged 1 cm below the surface of the water. Visual clues were positioned around the pool, to provide the mouse with spatial landmarks, and luminosity was maintained at 350 lux. The mice were initially exposed to a learning phase, which consisted of daily sessions (three trials per session) on five consecutive days. The starting position was varied pseudorandomly, between the four cardinal points. A mean interval of 15 min was left between trials. The trial was considered to have ended when the animal reached the platform. A 60-s cutoff was used, after which the mice were gently guided to the platform. Once on the platform, the animals were allowed to rest for 30 s before being returned to their cage. Long-term spatial memory was assessed 72 h after the last training trial (fifth day), in a probe trial in which the platform was no longer available. Animals were monitored with ANY-maze video tracking software (Stoelting Co, Wood Dale, USA).

### *Ex vivo* electrophysiology

Mice were anesthetized with halothane and decapitated. The brain was rapidly removed from the skull and placed in chilled (0–3 °C) artificial cerebrospinal fluid (ACSF) containing 124 mM NaCl, 3.5 mM KCl, 1.5 mM MgSO_4_, 2.5 mM CaCl_2_, 26.2 mM NaHCO_3_, 1.2 mM NaH_2_PO_4_, 11 mM glucose. Transverse slices (300–400 μm thick) were cut with a vibratome and placed in ACSF in a holding chamber, at 27 °C, for at least one hour before recording. Each slice was individually transferred to a submersion-type recording chamber and submerged in ACSF continuously superfused and equilibrated with 95 % O_2_, 5 % CO_2_.

The biophysical properties of the tonic current generated by the activation of extrasynaptic NMDA receptors with ambient glutamate were evaluated. Whole-cell patch-clamp recordings of CA1 pyramidal cells were performed at room temperature, with borosilicate patch pipettes (5 MΩ) filled with 140 mM CsCH_4_O_3_S, 6 mM CsCl, 2 mM MgCl_2_, 10 mM HEPES, 1.1 mM EGTA, 5 mM QX-314 5, 4 mM ATP, (pH 7.3; 290 mosm). Transmembrane currents were acquired and filtered through an amplifier (AxoPatch 1-D, Axon Instruments), stored on a computer and digitized with WinLTP software for analysis [62]. The tonic current was recorded at a holding potential of +40 mV, in the presence of TTX (1 μM), NBQX (10 μM), and bicuculline (10 μM), to isolate the NMDA component of the holding current (hc). After the recording of a stable control hc for three to five minutes, APV (50 μM) was added to the superfusion medium. The hc fell to a new stable value under the effect of APV, and the difference between the control hc and that recorded in the presence of APV determined the amplitude of the tonic current.

Two kinds of electrically induced long-term potentiation (LTP) were studied: a strong, saturating LTP consisting of 3x100 Hz (3x 100 pulses, 1 s, with 20 s between pulses), and a weaker stimulation, theta-burst stimulation (TBS), mimicking the natural stimulation at the theta frequency from the medial septum to the hippocampus, consisting of five trains of four 100 Hz pulses each, separated by 200 ms and delivered at the test intensity. The sequence was repeated three times, with an interburst interval of 10s. Testing with a single pulse was resumed for 60 min (TBS) or 75 min (3x100 Hz), to determine the level of LTP.

### Proton magnetic resonance spectroscopy (^1^H-MRS)

Magnetic Resonance Imaging and Magnetic Resonance Spectroscopy were performed with a horizontal 11.7 T scanner (Bruker, Ettlingen, Germany) and a quadrature cryoprobe was used for radiofrequency transmission and reception. A 37.4 μl voxel (7.2 × 2 × 2.6 mm^3^) was placed over both hemispheres, such that it contained essentially hippocampal tissue (Fig. 5), and the signal of this voxel was then averaged over 10 min. T_2_-weighted images were acquired with a 2D TurboRARE sequence (80 × 80 μm^2^ in-plane resolution, and 300 μm slice thickness) and manually segmented to measure hippocampal volume. ^1^H-MRS acquisitions were performed with a LASER sequence, with echo time (TE)/repetition time (TR) = 20/5000 ms and a 10 kHz bandwidth for the hyperbolic secant pulses. LCModel was used to determine metabolite concentrations. The macromolecule (MM) spectrum of a control mouse was determined by metabolite nulling and included in the base set for LCModel. The following metabolites were systematically quantified (Cramér-Rao lower limits <5 % in all experiments): total choline (glycerophosphocholine + phosphocholine + choline, tCho), total creatine (creatine + phosphocreatine, tCr), glutamate (Glu), glutamine (Gln), myo-inositol (Ins), N-acetyl-aspartate + N-acetyl-aspartyl-glutamate (NAA + NAAG, tNAA), taurine (T) and γ-aminobutyric acid (GABA). Metabolite concentrations were normalized with respect to 8 mM tCr.

### Statistical analysis

Data are expressed as mean ± SEM. Statistical analyses were performed with GraphPad Prism (GraphPad Software, La Jolla, CA, USA) or Statistica (StatSoft, Inc., Tulsa, OK, USA) software. One-way ANOVA and Tukey’s *post-hoc* test were used to determine the significance of differences between groups. Student’s *t* test was used when only two groups were analyzed (AAV-PS1 vs AAV-APP/PS1), except for tonic glutamatergic current recording, for which Chi^2^ tests were used. Two-way ANOVA with repeated measure using Group and Metabolite as effect factors, was used for NMR spectroscopy analysis.

## Additional files

Additional file 1: Figure S1. APP is processed in sporadic AD cases. Human samples were obtained from late-onset AD cases (Braak 6, Thal 5) and age-matched controls (*n* = 5 per group). The hippocampus was the studied structure. (**A**) Western blot analysis of NeuN. (**B**) Densitometric analyses of western blots showing the expression of human APP in the hippocampus of human controls and AD cases (*n* = 5 per group). Bars represent means ± SEM, and data were normalized with respect to GAPDH. Statistical analyses were performed with Student’s t-test: ***p* < 0.01. (**C**) Densitometric analyses of **A**, showing the expression of NeuN in the hippocampus of human controls and AD cases (*n* = 5 per group). Bars represent means ± SEM, and data were normalized with respect to GAPDH. Statistical analyses were performed with Student’s t-test: **p* < 0.05. (**D**) Representation of the APP/NeuN ratio following densitometric analyses of the corresponding western blots (*n* = 5 per group). Note that APP seems to be processed in sporadic AD cases. Bars represent means ± SEM and data were normalized with respect to GAPDH. Statistical analyses were performed with Student’s t-test: **p* < 0.05. (PNG 325 kb) Additional file 2: Figure S2. AAV-PS1 and AAV-APP mice do not exhibit neuronal defects in terms of PSD-95, GLT-1 and tonic glutamatergic current. C57Bl/6 J mice (all males) were injected at 8 weeks of age either with AAV-CAG-PS1M146L (AAV-PS1 mice) or AAV-CAG-APPSL (AAV-APP mice). Mice were killed three months later for analyses. (**A**) Western blot of PSD-95 and GLT-1 (*n* = 3-4 per group). (**B**) Densitometric analyses of the antibody immunoreactivities shown in panel **A**. (**C**) Tonic glutamatergic current recorded at a holding potential of +40 mV by the whole-cell patch-clamping of CA1 pyramidal cells. No significant difference in tonic glutamatergic current intensity was observed between the AAV-PS1 and AAV-APP groups (whole cell patch-clamp of CA1 pyramidal cells, *n* = 11/group from *n* = 10 mice per group). (PNG 447 kb)

## Abbreviations

AAV: Adeno-associated virus
Aβ: β-amyloid peptide
AD: Alzheimer’s disease
APP: Amyloid precursor protein
βCTF: β-carboxy-terminal fragment
ELISA: Enzyme-linked immunosorbent assay
NMDAR: N-methyl-D-aspartate receptor
PS1: Presenilin 1
