# Supplementary figures and images for: Alzheimer’s disease-like APP processing in wild-type mice identifies synaptic defects as initial steps of disease progression
Source: Mol Neurodegener. 2016 Jan 12;11:5. doi: 10.1186/s13024-016-0070-y (PMC4709894; doi:10.1186/s13024-016-0070-y)

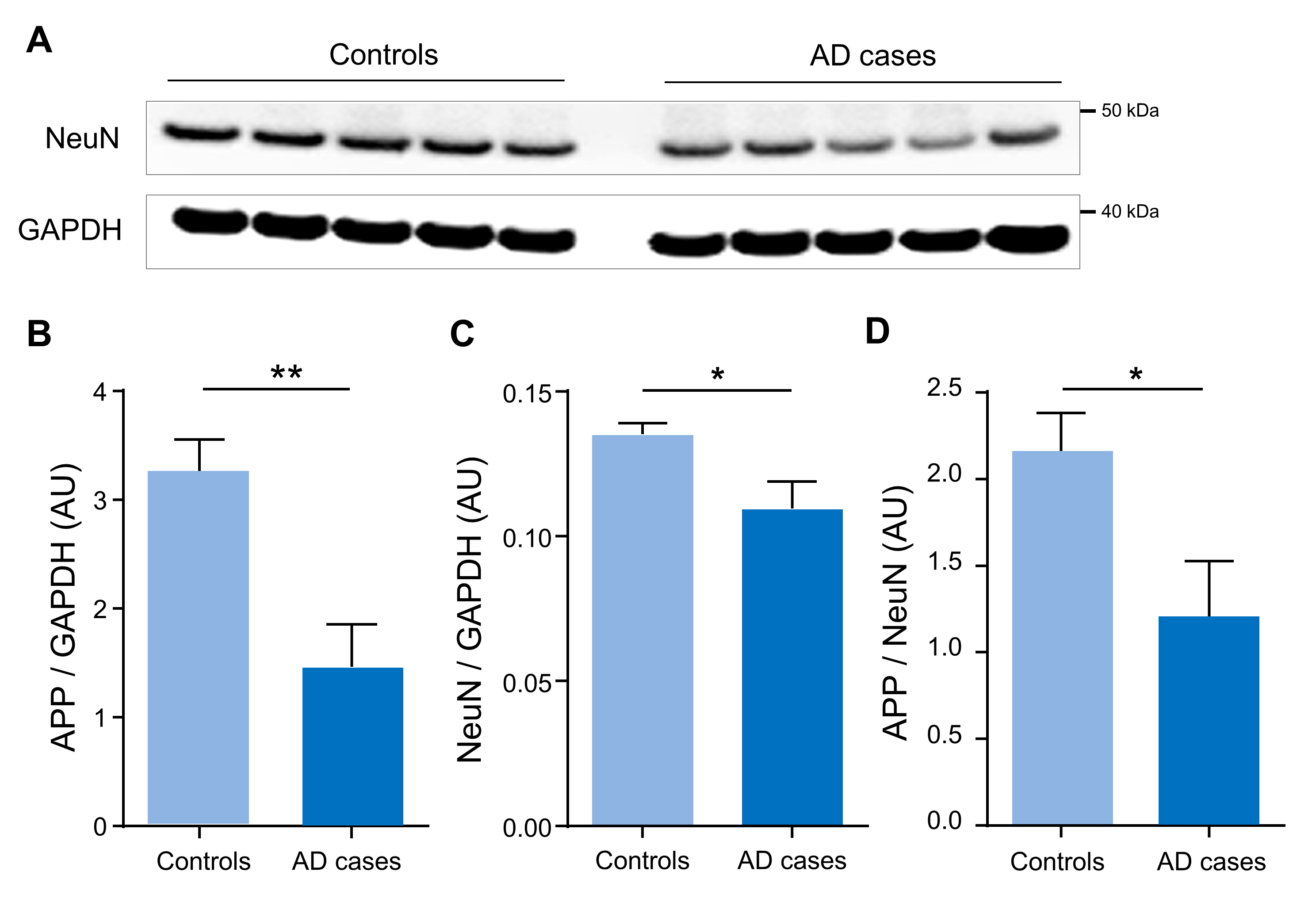

Supplement: Additional file 1: Figure S1. — APP is processed in sporadic AD cases. Human samples were obtained from late-onset AD cases (Braak 6, Thal 5) and age-matched controls (n = 5 per group). The hippocampus was the studied structure. (A) Western blot analysis of NeuN. (B) Densitometric analyses of western blots showing the expression of human APP in the hippocampus of human controls and AD cases (n = 5 per group). Bars represent means ± SEM, and data were normalized with respect to GAPDH. Statistical analyses were performed with Student’s t-test: **p < 0.01. (C) Densitometric analyses of A, showing the expression of NeuN in the hippocampus of human controls and AD cases (n = 5 per group). Bars represent means ± SEM, and data were normalized with respect to GAPDH. Statistical analyses were performed with Student’s t-test: *p < 0.05. (D) Representation of the APP/NeuN ratio following densitometric analyses of the corresponding western blots (n = 5 per group). Note that APP seems to be processed in sporadic AD cases. Bars represent means ± SEM and data were normalized with respect to GAPDH. Statistical analyses were performed with Student’s t-test: *p < 0.05. (PNG 325 kb) [file 13024_2016_70_MOESM1_ESM.png]

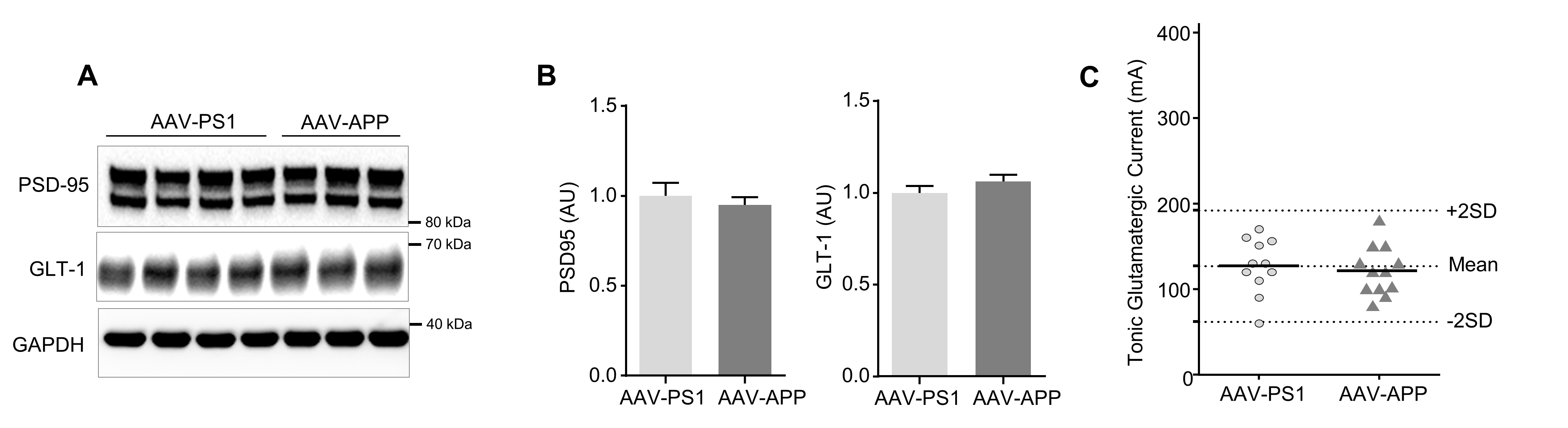

Supplement: Additional file 2: Figure S2. — AAV-PS1 and AAV-APP mice do not exhibit neuronal defects in terms of PSD-95, GLT-1 and tonic glutamatergic current. C57Bl/6 J mice (all males) were injected at 8 weeks of age either with AAV-CAG-PS1M146L (AAV-PS1 mice) or AAV-CAG-APPSL (AAV-APP mice). Mice were killed three months later for analyses. (A) Western blot of PSD-95 and GLT-1 (n = 3-4 per group). (B) Densitometric analyses of the antibody immunoreactivities shown in panel A. (C) Tonic glutamatergic current recorded at a holding potential of +40 mV by the whole-cell patch-clamping of CA1 pyramidal cells. No significant difference in tonic glutamatergic current intensity was observed between the AAV-PS1 and AAV-APP groups (whole cell patch-clamp of CA1 pyramidal cells, n = 11/group from n = 10 mice per group). (PNG 447 kb) [file 13024_2016_70_MOESM2_ESM.png]
